# (Epi)genetics of pregnancy-associated diseases

**DOI:** 10.3389/fgene.2013.00180

**Published:** 2013-09-10

**Authors:** Marie van Dijk, Cees Oudejans

**Affiliations:** ^1^Molecular Biology Laboratory, Department of Clinical Chemistry, VU University Medical CenterAmsterdam, Netherlands; ^2^Institute for Cardiovascular Research VU, VU University Medical CenterAmsterdam, Netherlands

**Keywords:** pre-eclampsia, HELLP, trophoblast, placenta, genetics, epigenetics, imprinting

## Abstract

This review describes the current knowledge regarding genetics and epigenetics of pregnancy-associated diseases with placental origin. We discuss the effect on genetic linkage analyses when the fetal genotype determines the maternal phenotype. Secondly, the genes identified by genome-wide linkage studies to be associated with pre-eclampsia (*ACVR2A*, *STOX1*) and the HELLP-syndrome (*LINC-HELLP*) are discussed regarding their potential functions in the etiology of disease. Furthermore, susceptibility genes identified by candidate gene approaches (e.g., *CORIN*) are described. Next, we focus on the additional challenges that come when epigenetics also play a role in disease inheritance. We discuss the maternal transmission of the chromosome 10q22 pre-eclampsia linkage region containing the *STOX1* gene and provide further evidence for the role of epigenetics in pre-eclampsia based on the *cdkn1c* mouse model of pre-eclampsia. Finally, we provide recommendations to unravel the genetics of pregnancy-associated diseases, specifically regarding clear definitions of patient groups and sufficient patient numbers, and the potential usefulness of (epi)genetic data in early non-invasive biomarker development.

## INTRODUCTION

This review will describe the current status of the genetics and epigenetics behind pregnancy-associated diseases with placental origin. It focuses especially on the manner of inheritance of these maternal diseases that originate in the fetal placenta as this complicates genome-wide association studies. The second focus centers on the additional challenges that come into play when epigenetics are also involved in the inheritance of disease.

Pregnancy-induced hypertension, pre-eclampsia, eclampsia and the HELLP syndrome are all diseases that occur during pregnancy giving symptoms in the mother from 20 weeks of gestation onward. Next to pregnancy-induced hypertension also pre-eclampsia is characterized by hypertension occurring simultaneously with proteinuria. In eclampsia patients suffer from seizures, while in the HELLP syndrome the symptoms consist of haemolysis, elevated liver enzymes and low platelet count (Steegers et al., 2010). Complete clinical characteristics including the occurrence percentages can be found in **Table 1**. The only cure currently available for all of these diseases is removal of the placenta and thus delivery of the baby.

**Table 1:** Clinical subtypes of pregnancy-induced hypertension.

Disease	Clinical symptoms^*^	Occurrence^#^
Pregnancy-induced hypertension	Blood pressure >140/90	6–8%
Pre-eclampsia	Blood pressure >140/90	5–8%
	Proteinuria >300 mg/24 h	
Eclampsia	Blood pressure >140/90	0.05%
	Tonic-clonic seizures	
HELLP syndrome	LD > 600 IU/l	0.5–1%
	ASAT > 70 IU/l	
	ALAT > 70 IU/l	
	Platelets <100 × 10^9^/l	

* Steegers etal., 2010

# World Health Organisation (2005), Wallis etal., 2008, Duley (2009), Haram etal., 2009.

The pregnancy-associated diseases with placental origin are seen both separately and in combination, e.g., eclampsia and the HELLP syndrome are often superimposed on pre-eclampsia. However, it gets more widely acknowledged that the diseases are different entities. Furthermore, it is important to stress that a differentiation is required between early- and late-onset of disease, where early-onset is defined as the occurrence of clinical symptoms before 34 weeks. Early-onset is furthermore characterized by a high risk of fetal and maternal morbidity and mortality, an abnormal placental morphology, a clear familial component and association with intra-uterine growth restriction (defined as a decrease in fetal growth rate that prevents an infant from obtaining its complete growth potential). On the other hand, late-onset disease shows normal placental morphology, but is characterized by many maternal risk factors such as diabetes, cardiovascular disorders, increased body mass index (BMI), etc (Steegers et al., 2010).

## GENETICS OF PRE-ECLAMPSIA AND THE HELLP SYNDROME

### FETAL GENOTYPE DETERMINES MATERNAL PHENOTYPE

Most (genetic) research investigating pregnancy-associated diseases is performed on the early-onset forms of disease. These forms not only have the highest morbidity and mortality risk for both mother and child, but, as these forms run in families, also have a genetic component (Laivuori, 2007). The consistent observations that the early-onset form shows an abnormal placental morphology and is often associated with intra-uterine growth restriction indicate that early-onset disease originates in the fetal placenta (Roberts and Hubel, 2009). During a healthy pregnancy, the fetal-maternal interface is formed in the first trimester of pregnancy when extravillous trophoblasts from the fetal placenta invade into the maternal decidua up to one-third of the myometrium. By invading the maternal decidua, the maternal spiral arteries located within the decidua are remodeled, transforming them from low-capacity high-resistance into high-capacity low-resistance vessels, by replacing smooth muscle and elastic tissue with fibrinoid material (James et al., 2010).

In early-onset pregnancy-associated disease a reduction in fetal trophoblast invasion is observed together with reduced spiral artery remodeling. This in turn decreases the blood flow toward the placenta reducing the oxygen and nutrient transfer to the fetus. To compensate, the mother increases her blood pressure, at the end of the second and in third trimester leading to the symptoms as described above (James et al., 2010).

As early-onset disease originates in the fetal placenta it must be concluded that the fetal genotype of the placental trophoblasts is (partially) able to determine the maternal disease phenotype. If pre-eclampsia is considered to be an autosomal recessive disease, the father and mother are genetic carriers and their unborn child with its placenta is genetically affected, leading to the phenotypic disease in the mother. Further evidence for the placental origin of early-onset pre-eclampsia comes from the consistent observation that parous monozygotic twins are discordant for pre-eclampsia (Treloar et al., 2001).

It is now widely accepted that pre-eclampsia is a multi-factorial disease in which both maternal and fetoplacental factors are contributing; different combinations of factors will lead to differences in time of onset, disease severity, etc. Some of these factors will be hereditary leading to the familial components seen in early-onset pregnancy-associated diseases. The first step in identifying the fetal and maternal causal factors is to subcategorize the disease to yield homogenous patient populations. Secondly, when performing research on genetic linkage in familial early-onset disease originating in the placenta, the study should focus on the placental genotype rather than the maternal genotype. This implies it is a necessity to have samples from both the mother and the child born out of the affected pregnancy. Another option is to consider an affected mother born from an unaffected mother carrier, while affected mothers born from affected pregnancies themselves can be considered cases.

### SUSCEPTIBILITY GENES IN PRE-ECLAMPSIA

To identify genes involved in pre-eclampsia multiple genetic screens (genome-wide scans, variance-components linkage analysis, association analysis) have been performed in multiple patient populations (families, case-control; Arngrímsson et al., 1999; Moses et al., 2000; Lachmeijer et al., 2001; Laasanen et al., 2003; Laivuori et al., 2003; Fitzpatrick et al., 2004; Oudejans et al., 2004; Johnson et al., 2007). This has so far yielded limited results with only two susceptibility genes (*ACVR2A* and *STOX1*) identified (Van Dijk et al., 2005; Moses et al., 2006), within confirmed regions with significant genome-wide linkage. For both susceptibility genes the alleles associated involve normal variations (SNPs: single nucleotide polymorphisms) consistent with the common variant-common disease hypothesis (Wang et al., 2005). Furthermore, both were originally identified in familial forms of pre-eclampsia.

#### ACVR2A

*ACVR2A* is located on chromosome 2q22 and was originally identified in an Australian/New Zealand population of pre-eclamptic pedigrees (Moses et al., 2006). This linkage was subsequently confirmed in a Norwegian case-control study (Roten et al., 2009), although later studies in a subset of the Australian population (Fitzpatrick et al., 2009) as well as a Finnish population (Lokki et al., 2011) were unable to identify significant linkage. The *ACVR2A* gene codes for an Activin receptor type II that is able to bind Activin ligands. Activin A has a role on both the maternal and the fetal site of the placenta, where on the maternal site it promotes decidualization of endometrial stromal cells and regulates trophoblast invasion into the decidua (Jones et al., 2002), while on the fetal site it promotes trophoblast differentiation (Caniggia et al., 1997). Furthermore, multiple studies have found increased Activin A levels in serum of pre-eclamptic women (Giguère et al., 2010). None of the SNPs identified by *ACVR2A* linkage are located in exons. They therefore might not appear to be relevant in the etiology of pre-eclampsia, but they are potentially located in areas controlling *ACVR2A* transcription or regulation. For instance, if the SNP is located within the promoter region of *ACVR2A* it can affect the binding of transcription factors regulating transcription of the gene or, when the SNP for example is located within a microRNA binding site, it might affect the binding of microRNAs causing a change in the amount of *ACVR2A* mRNA available for translation into protein.

#### STOX1

*STOX1* was originally identified in a Dutch population consisting of affected siblings and their relatives, in which the affected women showed familial severe early-onset pre-eclampsia complicated by IUGR (intra uterine growth restriction). The coding linkage regions on chromosome 10q22 were sequenced identifying a common polymorphism in *STOX1*, *Y153H*, with maternal transmission in three generations (Van Dijk et al., 2005). Linkage of this gene to pre-eclampsia has also been difficult to replicate in other population cohorts (Iglesias-Platas et al., 2007; Kivinen et al., 2007). This is mainly due to unclearly defined disease characteristics of the pre-eclamptic women; the populations consisted of a combination of mild and severe, early- and late-onset pre-eclampsia, and the disease did not always show familial inheritance. Furthermore, as the replication studies were performed on maternal genotypes, this implies another method needs to be used for linkage data analysis to correct for the fetal genotype determining the maternal phenotype as described above. One study, also performed in a Dutch population, did take both criteria, i.e., a homogenous patient population and familial inheritance enabling to correct for the fetal genotype determining the maternal phenotype, into account; using a homogenous patient population and three generations with a grandmaternal origin of the disease, a significant maternal transmission of the *STOX1 Y153H* allele was found (Berends et al., 2007). A study in a Norwegian population could not find a significant transmission of the pre-eclampsia susceptibility H-allele, but, when analyzing recurrent pre-eclampsia patients only (where disease recurrence is indicative of genetic origin), a trend toward significance of maternal transmission was detected (Fenstad et al., 2010).

The *STOX1 *gene codes for a protein containing a winged helix domain, indicating that it acts as a transcription factor (Van Dijk et al., 2005). The Y153H SNP is located within the DNA binding winged helix domain and the H-allele, the allele associated with pre-eclampsia, has been found to have a higher binding affinity for *CTNNA3*, a gene transcribed by binding of STOX1 (Van Dijk et al., 2010). *CTNNA3*, coding for αT-catenin, is a component of the cell–cell adhesion complex (Janssens et al., 2001). Upregulation of αT-catenin causes a reduction in invasion capacity of trophoblasts (Tyberghein et al., 2012), while proliferation is promoted (Van Dijk et al., 2010). First trimester placental explants homozygous for the *STOX1* H-allele showed reduced trophoblast invasion and induced responsiveness of *CTNNA3* expression upon *STOX1* expression (Van Dijk et al., 2010). This genotype, STOX1 Y153H, therefore has a demonstrated effect on trophoblast invasion, central in the etiology of early-onset pre-eclampsia.

By overexpressing STOX1 in human choriocarcinoma cells transcriptomic alterations were induced that resembled those seen in pre-eclamptic placentas (Rigourd et al., 2008). Recently, this group developed transgenic mouse strains overexpressing human STOX1 (Doridot et al., 2013). Wildtype female mice crossed with transgenic male mice developed severe pre-eclamptic symptoms, i.e., gestational hypertension and proteinuria. Furthermore, the placenta and kidney morphologies were altered. As the mouse pregnancies were in a wildtype female, the disease in these mice confirms a fetoplacental origin of severe early-onset pre-eclampsia caused in these mice by human STOX1 over-expression.

### SUSCEPTIBILITY GENE FOR THE HELLP-SYNDROME IS A LONG NON-CODING RNA

The original Dutch study in families affected by severe pre-eclampsia also consisted of families affected by the HELLP-syndrome. Re-analysis of the original genome-wide scan performed in these families led to the conclusion that in families without the HELLP-syndrome (pre-eclampsia families) different chromosomal linkage regions could be identified as were found in the families with HELLP-syndrome (Lachmeijer et al., 2001). This indicated that pre-eclampsia and the HELLP-syndrome are different genetic entities. The chromosomal linkage associated with the HELLP-syndrome has recently been found to be located on chromosome 12q23 (Van Dijk et al., 2012). Here, a long non-coding RNA (205 kb) has been identified that is expressed in extravillous trophoblasts of first trimester placenta. Furthermore, knockdown of this long non-coding RNA activated a large set of genes involved in the cell cycle, associated with a reduced activity of the G2/M phase while the G1/S phase activity was increased, together suggesting a reduction in proliferative activity of the cells. Finally, by blocking mutation sites, as identified in children born out of HELLP pregnancies, transcription of the non-coding RNA itself was upregulated causing a reduction in the amount of trophoblast cell invasion. This would fit the placental origin of the HELLP-syndrome in which extravillous trophoblasts show reduced invasion into the maternal decidua, caused by reduced differentiation of the extravillous trophoblasts from a proliferative to an invasive phenotype, already indicated by the cell cycle effects mediated by siRNA knockdown.

## CANDIDATE GENE APPROACHES IN PRE-ECLAMPSIA

Next to genetic screens also the candidate gene approach has been used to detect genes associated with, primarily, pre-eclampsia. These screens have largely focused on the maternal genotypes involved and choose a single gene to investigate, based on biological function and thus potential involvement in the pathology of pre-eclampsia. Pathways that have been under investigation include pathways involved in oxidative stress, endothelial injury, vasoactivity, coagulation, lipid metabolism, and inflammation. The major problem with this type of research is again the differences in patient populations used. Furthermore, because the patients do not have a familial component, the number of patients needed to identify susceptibility genes is much higher and therefore never reached to obtain reproducible results. The numbers of genes that have been studied are too many to describe in this review, but a recent meta-analysis identified seven genetic variants to be consistently associated with pre-eclampsia (Buurma et al., 2013). These were located in or near the genes *ACE*, *CTLA4*, *F2*, *FV* (two variants), *LPL,* and *SERPINE1*. The functional significance of these variants in pre-eclampsia however yet needs to be established.

Recently, a maternal pre-eclampsia susceptibility gene has been described that influences trophoblast invasion and spiral artery remodeling, i.e., *CORIN *(Cui et al., 2012). Corin is a cardiac protease that activates atrial natriuretic peptide (ANP), a cardiac hormone that is involved in blood pressure regulation. Corin- or ANP-deficient mice developed pre-eclampsia-like symptoms. Furthermore, it was found that pre-eclamptic patients had reduced uterine Corin levels. Finally, Corin gene mutations were identified in pre-eclamptic patients leading to decreased corin activity. How common mutations in this gene are in pre-eclamptic patients remains to be investigated.

An interesting immunological fetal-maternal genetic interaction should also be noted; HLA-C is a polymorphic gene expressed by the invasive extravillous trophoblasts and a ligand for killer immunoglobulin-like receptors (KIRs) that are expressed on the maternal uterine natural killer cells (uNK). KIRs have two different alleles, A and B. It has been found that mothers that have the AA KIR genotype while the fetus has HLA-C belonging to the HLA-C2 group have an increased risk of developing pre-eclampsia (Chazara et al., 2011).

## EPIGENETICS OF PRE-ECLAMPSIA

### EPIGENETICS

In genetics the term epigenetics is widely used these days to describe heritable changes that are not caused by changes in the underlying DNA sequence. The modifications mainly consist of DNA methylation and histone modifications, both of which are able to regulate gene expression. These changes are heritable, i.e., they are maintained through multiple cell divisions.

A specific type of epigenetics is imprinting. During imprinting only the allele inherited from mother or father is subject to modifications to regulate gene expression. This has implications for the inheritance of disease; it will behave as an autosomal dominant disease when inherited from the non-imprinted parent, while it will be silent when inherited from the imprinted parent. It becomes even more complicated when the concept of fetal genotype determines maternal phenotype is introduced. In case of a paternally imprinted, maternally expressed gene, the child born from a phenotypically diseased pregnancy only needs to have inherited the genotypically diseased allele from the mother (being homozygous or heterozygous herself) to give the mother symptoms during pregnancy. When the father is heterozygous or homozygous for the diseased allele the pregnancy will not be affected.

### MATERNAL TRANSMISSION OF THE 10q22 CHROMOSOMAL REGION

The genome-wide scan using microsatellite markers in the Dutch pre-eclampsia families already exposed a parent-of-origin effect; only the maternal allele seemed to be involved in the linkage observed (Oudejans et al., 2004). By sequencing the complete linkage region on DNA of multiple generations, identifying *STOX1 Y153H*, maternal transmission of the *Y153H* allele was found (Van Dijk et al., 2005). However, no differential methylation could be detected in the CpG island of the promoter region of *STOX1*, the area predominantly affected by methylation leading to differential expression (Van Dijk et al., 2007). A second CpG island was identified in intron 1 of *STOX1*. Methylation of this CpG island led to reduced expression consistent with the normal association between hypermethylation and downregulation of expression (Van Dijk et al., 2010). Furthermore, in specific cell types, i.e., proliferating extravillous trophoblasts, this region was subject to differential and allele-specific methylation, where the methylated allele was paternal in origin. Early placenta samples consisting of multiple cell types, no allele-specific methylation could be detected, but an overall increase in methylation was seen in samples homozygous for the *STOX1 Y153H* allele. As proliferating extravillous trophoblasts are differentiated from villous cytotrophoblasts, it is hypothesized that these cells are paternally imprinted as well. In cell types where methylation is independent of parental origin, the Y153H genotype has the potential to direct the level of methylation.

### *cdkn1c* PRE-ECLAMPSIA MICE

*CDKN1C* is paternally imprinted in both humans and mice. Pregnant mice heterozygous for *cdkn1c* deficiency (-/+) display pre-eclampsia-like symptoms, i.e., hypertension and proteinuria. This occurred in female -/+ mice that were mated with -/+ males or wildtype males, but not when wildtype females were mated with -/+ males. The pregnant -/+ mice carried conceptuses with and without* cdkn1c* expression, only the conceptuses without *cdkn1c* expression showed a lack of trophoblast invasion and increase in trophoblast proliferation in the placenta, suggested to cause the maternal symptoms (Kanayama et al., 2002). The human *CDKN1C* gene does not show linkage with pre-eclampsia, making its role in human pre-eclampsia unknown. However, the importance of epigenetics in pregnancy-associated diseases is clear.

## FUTURE DIRECTIONS

Pregnancy-associated diseases with placental origin, especially pre-eclampsia, are multi-factorial diseases in which both maternal and fetoplacental factors are contributing, where different combinations of factors will lead to differences in disease characteristics. Some of these factors will be hereditary giving rise to the familial component of the disease.

There is still a long way to go before the genetics of these diseases are unraveled. This has a number of causes; the first problem lies in the definition of different patient groups. As long as patients (to identify maternal genes), and children born from diseased pregnancies (to identify fetal genes), are not clearly defined and separated according to time of onset, placental morphology, familial components, association with e.g., IUGR and associations with maternal risk factors such as diabetes, cardiovascular disorders, increased BMI, etc., it is almost impossible to identify the susceptibility genes involved. Secondly, and this is especially the case when investigating non-familial cases, the number of patients needs to be extremely high to have enough power to identify variations between patients and controls as the diseases are so heterogeneous.

When more is known on the (epi)genetics of pregnancy-associated diseases it will be easier to develop early non-invasive diagnostic tests to be able to perform accurate risk profiling. Preferably these tests are performed in maternal plasma, which also contains fetal DNA and RNA, obtained in the first trimester of pregnancy before the onset of symptoms in the mother.

Using maternal plasma to detect fetal DNA and RNA is complicated by the fact that 90–95% of the plasma consists of nucleic acids of maternal origin. To be able to discriminate between maternal and fetal DNA for epigenetic analysis it is therefore important to only use chromosomal regions that are found to be either highly methylated (>80%) or highly unmethylated (<20%) when analyzed in plasma of non-pregnant women. Furthermore, to properly discriminate between fetal and maternal DNA the difference in methylation levels between non-pregnant and pregnant plasma should be at least 50%. When analyzing RNA expression differences between pregnant patients and healthy pregnant controls, the expression should preferably be completely absent in non-pregnant plasma samples. Although the only cure currently available is delivery of the baby, early diagnosis will increase monitoring of women at risk and thereby decreasing morbidity and mortality in both mother and child.

Unraveling the underlying (epi)genetic causes of pregnancy-associated diseases with placental origin will not only yield options for early diagnostics but will also gain fundamental insight into the origin of disease, providing options for prevention and therapeutic intervention.

## Conflict of Interest Statement

The authors declare that the research was conducted in the absence of any commercial or financial relationships that could be construed as a potential conflict of interest.

## References

[B1] Arngrímsson R. Sigurõardóttir S. Frigge M. L. Bjarnadóttir R. I. Jónsson T. Stefánsson H. (1999). A genome-wide scan reveals a maternal susceptibility locus for pre-eclampsia on chromosome 2p13. *Hum. Mol. Genet.* 8 1799–1805 10.1093/hmg/8.9.1799 10441346

[B2] Berends A. L. Bertoli-Avella A. M. de Groot C. J. van Duijn C. M. Oostra B. A. Steegers E. A. (2007). STOX1 gene in pre-eclampsia and intrauterine growth restriction. *BJOG* 114 1163–1167 10.1111/j.1471-0528.2007.01414.x 17617193

[B3] Buurma A. J. Turner R. J. Driessen J. H. Mooyaart A. L. Schoones J. W. Bruijn J. A. (2013). Genetic variants in pre-eclampsia: a meta-analysis. *Hum. Reprod. Update* 19 289–303 10.1093/humupd/dms060 23300202

[B4] Caniggia I. Lye S. J. Cross J. C. (1997). Activin is a local regulator of human cytotrophoblast cell differentiation. *Endocrinology* 138 3976–3986 10.1210/en.138.9.3976 9275089

[B5] Chazara O. Xiong S. Moffett A. (2011). Maternal KIR and fetal HLA-C: a fine balance. *J. Leukoc. Biol.* 90 703–716 10.1189/jlb.0511227 21873457

[B6] Cui Y. Wang W. Dong N. Lou J. Srinivasan D. K. Cheng W. (2012). Role of corin in trophoblast invasion and uterine spiral artery remodelling in pregnancy. *Nature* 484 246–250 10.1038/nature10897 22437503 PMC3578422

[B7] Doridot L. Passet B. Méhats C. Rigourd V. Barbaux S. Ducat A. (2013). Preeclampsia-like symptoms induced in mice by fetoplacental expression of STOX1 are reversed by aspirin treatment. *Hypertension* 61 662–668 10.1161/HYPERTENSIONAHA.111.202994 23357179

[B8] Duley L. (2009). The global impact of pre-eclampsia and eclampsia. *Semin. Perinatol.* 33 130–137 10.1053/j.semperi.2009.02.010 19464502

[B9] Fenstad M. H. Johnson M. P. Løset M. Mundal S. B. Roten L. T. Eide I. P. (2010). STOX2 but not STOX1 is differentially expressed in decidua from pre-eclamptic women: data from the Second Nord-Trondelag Health Study. *Mol. Hum. Reprod.* 16 960–968 10.1093/molehr/gaq064 20643876 PMC2989830

[B10] Fitzpatrick E. Göring H. H. Liu H. Borg A. Forrest S. Cooper D. W. (2004). Fine mapping and SNP analysis of positional candidates at the preeclampsia susceptibility locus (PREG1) on chromosome 2. *Hum. Biol.* 76 849–862 10.1353/hub.2005.0017 15974297

[B11] Fitzpatrick E. Johnson M. P. Dyer T. D. Forrest S. Elliott K. Blangero J. (2009). Genetic association of the activin A receptor gene (ACVR2A) and pre-eclampsia. *Mol. Hum. Reprod.* 15 195–204 10.1093/molehr/gap001 19126782 PMC2647107

[B12] Giguère Y. Charland M. Bujold E. Bernard N. Grenier S. Rousseau F. (2010). Combining biochemical and ultrasonographic markers in predicting preeclampsia: a systematic review. *Clin. Chem.* 56 361–375 10.1373/clinchem.2009.134080 20044446

[B13] Haram K. Svendsen E. Abildgaard U. (2009). The HELLP syndrome: clinical issues and management. A Review. *BMC Pregnancy Childbirth* 26:8 10.1186/1471-2393-9-8 PMC2654858 19245695

[B14] Iglesias-Platas I. Monk D. Jebbink J. Buimer M. Boer K. van der Post J. (2007). STOX1 is not imprinted and is not likely to be involved in preeclampsia. *Nat. Genet.* 39 279–280 10.1038/ng0307-279 17325670

[B15] James J. L. Whitley G. S. Cartwright J. E. (2010). Pre-eclampsia: fitting together the placental, immune and cardiovascular pieces. *J. Pathol.* 221 363–378 10.1002/path.2719 20593492

[B16] Janssens B. Goossens S. Staes K. Gilbert B. van Hengel J. Colpaert C. (2001). alphaT-catenin: a novel tissue-specific beta-catenin-binding protein mediating strong cell-cell adhesion. *J. Cell. Sci.* 114 3177–3188 11590244 10.1242/jcs.114.17.3177

[B17] Johnson M. P. Fitzpatrick E. Dyer T. D. Jowett J. B. Brennecke S. P. Blangero J. (2007). Identification of two novel quantitative trait loci for pre-eclampsia susceptibility on chromosomes 5q and 13q using a variance components-based linkage approach. *Mol. Hum. Reprod.* 13 61–67 10.1093/molehr/gal095 17085769

[B18] Jones R. L. Salamonsen L. A. Findlay J. K. (2002). Activin A promotes human endometrial stromal cell decidualization in vitro. *J. Clin. Endocrinol. Metab.* 87 4001–4004 10.1210/jc.87.8.4001 12161551

[B19] Kanayama N. Takahashi K. Matsuura T. Sugimura M. Kobayashi T. Moniwa N. (2002). Deficiency in p57Kip2 expression induces preeclampsia-like symptoms in mice. *Mol. Hum. Reprod.* 8 1129–1135 10.1093/molehr/8.12.1129 12468647

[B20] Kivinen K. Peterson H. Hiltunen L. Laivuori H. Heino S. Tiala I. (2007). Evaluation of STOX1 as a preeclampsia candidate gene in a population-wide sample. *Eur. J. Hum. Genet.* 15 494–497 10.1038/sj.ejhg.5201788 17290274

[B21] Laasanen J. Hiltunen M. Romppanen E. L. Punnonen K. Mannermaa A. Heinonen S. (2003). Microsatellite marker association at chromosome region 2p13 in Finnish patients with preeclampsia and obstetric cholestasis suggests a common risk locus. *Eur. J. Hum. Genet.* 11 232–236 10.1038/sj.ejhg.5200951 12673277

[B22] Lachmeijer A. M. Arngrímsson R. Bastiaans E. J. Frigge M. L. Pals G. Sigurdardóttir S. (2001). A genome-wide scan for preeclampsia in the Netherlands. *Eur. J. Hum. Genet.* 9 758–764 10.1038/sj.ejhg.5200706 11781687

[B23] Laivuori H. (2007). Genetic aspects of preeclampsia. *Front. Biosci.* 12: 2372–2382 10.2741/2239 17127247

[B24] Laivuori H. Lahermo P. Ollikainen V. Widen E. Häivä-Mällinen L. Sundström H. (2003). Susceptibility loci for preeclampsia on chromosomes 2p25 and 9p13 in Finnish families. *Am. J. Hum. Genet.* 72 168–177 10.1086/345311 12474145 PMC378622

[B25] Lokki A. I. Klemetti M. M. Heino S. Hiltunen L. Heinonen S. Laivuori H. (2011). Association of the rs1424954 polymorphism of the ACVR2A gene with the risk of pre-eclampsia is not replicated in a Finnish study population. *BMC Res. Notes* 19:545 10.1186/1756-0500-4-545 PMC3267796 22177086

[B26] Moses E. K. Fitzpatrick E. Freed K. A. Dyer T. D. Forrest S. Elliott K. (2006). Objective prioritization of positional candidate genes at a quantitative trait locus for pre-eclampsia on 2q22. *Mol. Hum. Reprod.* 12 505–512 10.1093/molehr/gal056 16809377

[B27] Moses E. K. Lade J. A. Guo G. Wilton A. N. Grehan M. Freed K. (2000). A genome scan in families from Australia and New Zealand confirms the presence of a maternal susceptibility locus for pre-eclampsia, on chromosome 2. *Am. J. Hum. Genet.* 67 1581–1585 10.1086/316888 11035632 PMC1287935

[B28] Oudejans C. B. Mulders J. Lachmeijer A. M. van Dijk M. Könst A. A. Westerman B. A. (2004). The parent-of-origin effect of 10q22 in pre-eclamptic females coincides with two regions clustered for genes with down-regulated expression in androgenetic placentas. *Mol. Hum. Reprod.* 10 589–598 10.1093/molehr/gah080 15208369

[B29] Rigourd V. Chauvet C. Chelbi S. T. Rebourcet R. Mondon F. Letourneur F. (2008). STOX1 overexpression in choriocarcinoma cells mimics transcriptional alterations observed in preeclamptic placentas. *PLoS ONE* 3:e3905 10.1371/journal.pone.0003905 PMC2592700 19079545

[B30] Roberts J. M. Hubel C. A. (2009). The two stage model of preeclampsia: variations on the theme. *Placenta* 30(Suppl. A) S32–S37 10.1016/j.placenta.2008.11.009 19070896 PMC2680383

[B31] Roten L. T. Johnson M. P. Forsmo S. Fitzpatrick E. Dyer T. D. Brennecke S. P. (2009). Association between the candidate susceptibility gene ACVR2A on chromosome 2q22 and pre-eclampsia in a large Norwegian population-based study (the HUNT study). *Eur. J. Hum. Genet.* 17 250–257 10.1038/ejhg.2008.158 18781190 PMC2696227

[B32] Steegers E. A. von Dadelszen P. Duvekot J. J. Pijnenborg R. (2010). Pre-eclampsia. *Lancet* 376 631–644 10.1016/S0140-6736(10)60279-6 20598363

[B33] Treloar S. A. Cooper D. W. Brennecke S. P. Grehan M. M. Martin N. G. (2001). An Australian twin study of the genetic basis of preeclampsia and eclampsia. *Am. J. Obstet. Gynecol.* 184 374–381 10.1067/mob.2001.109400 11228490

[B34] Tyberghein K. Goossens S. Haigh J. J. van Roy F van Hengel J. (2012). Tissue-wide overexpression of alpha-T-catenin results in aberrant trophoblast invasion but does not cause embryonic mortality in mice. *Placenta* 33 554–560 10.1016/j.placenta.2012.04.002 22534068

[B35] Van Dijk M. Drewlo S. Oudejans C. B. (2010). Differential methylation of STOX1 in human placenta. *Epigenetics* 5 736–742 10.4161/epi.5.8.13084 20716964

[B36] Van Dijk M. Mulders J. Poutsma A. Könst A. A. Lachmeijer A. M. Dekker G. A. (2005). Maternal segregation of the Dutch preeclampsia locus at 10q22 with a new member of the winged helix gene family. *Nat. Genet.* 37 514–519 10.1038/ng1541 15806103

[B37] Van Dijk M. Thulluru H. K. Mulders J. Michel O. J. Poutsma A. Windhorst S. (2012). HELLP babies link a novel lincRNA to the trophoblast cell cycle. *J. Clin. Invest.* 122 4003–4011 10.1172/JCI65171 23093777 PMC3484460

[B38] Van Dijk M. Van Bezu J. Chim S. S. Lo Y. M. Blankenstein M. A. Oudejans C. B. (2007). Reply to: STOX1 is not imprinted and is not likely to be involved in preeclampsia. *Nat. Genet.* 39 280–281 10.1038/ng0307-280 17325670

[B39] Van Dijk M. van Bezu J. van Abel D. Dunk C. Blankenstein M. A. Oudejans C. B. (2010). The STOX1 genotype associated with pre-eclampsia leads to a reduction of trophoblast invasion by alpha-T-catenin upregulation. *Hum. Mol. Genet.* 19 2658–2667 10.1093/hmg/ddq152 20400461

[B40] Wallis A. B. Saftlas A. F. Hsia J. Atrash H. K. (2008). Secular trends in the rates of preeclampsia, eclampsia, and gestational hypertension, United States, 1987-2004. *Am. J. Hypertens.* 21 521–526 10.1038/ajh.2008.20 18437143

[B41] Wang W. Y. Barratt B. J. Clayton D. G. Todd J. A. (2005). Genome-wide association studies: theoretical and practical concerns. *Nat. Rev. Genet.* 6 109–118 10.1038/nrg1522 15716907

[B42] World Health Organisation (2005). *The World Health Report (2005) – make every mother and child count*. Geneve: World Health Organisation

